# Impact of ChatGPT on Interdisciplinary Nursing Education and Research

**DOI:** 10.2196/48136

**Published:** 2023-04-24

**Authors:** Hongyu Miao, Hyochol Ahn

**Affiliations:** 1 Florida State University Tallahassee, FL United States

**Keywords:** ChatGPT, nursing education, nursing research, artificial intelligence, OpenAI

## Abstract

ChatGPT, a trending artificial intelligence tool developed by OpenAI, was launched in November 2022. The impact of ChatGPT on the nursing and interdisciplinary research ecosystem is profound.

ChatGPT (also known as Chat Generative Pretrained Transformer) is a trending artificial intelligence (AI) tool developed by OpenAI [1]. It was first launched in November 2022 based on OpenAI’s GPT-3.5 [2], followed by the second release shortly in March 2023 based on GPT-4.0 [3]. Two months after its first release, the number of active users per month reached over 100 million, making ChatGPT the fastest-growing consumer application ever [4]. Technically, ChatGPT is a large language model–based chatbot that performs specific natural language processing tasks. For people who believe in deep learning technologies, they will immediately tell from the name of ChatGPT that the powerfulness of this tool is largely attributed to the attention model introduced by a group of Google researchers in 2017 [5]; however, even among users who are new to AI, ChatGPT is still well accepted as its user interface is straightforward with all the complex technical details hidden. More importantly, it is almost omnipotent in terms of answering a wide variety of questions like a knowledgeable human being most of the time.

Training ChatGPT for its versatility and powerfulness is not cheap. According to miscellaneous information sources, OpenAI originally used ~40 GB of text data to train the early GPT model with 8 NVIDIA V100 GPUs and 256 GB of RAM. To train GPT-3, which laid the foundation for ChatGPT, the 2016-2019 Common Crawl data set [6] of 45 TB of compressed plain text was used. Nowadays the data set used for training ChatGPT consists of more than 145 million dialogues scraped from various social media and online knowledge bases (eg, Twitter, Reddit, and Wikipedia). Note that it is also expensive to clean up such text data as spam, offensive language, low-quality content, and so on need to be removed before they can be fed to ChatGPT. The typical hardware configuration for training ChatGPT may include 64 or more NVLink-connected NVIDIA V100 GPUs with 32 GB of memory each, and each round of training may take 2 weeks. The estimated cost of training ChatGPT is close to US $5 million dollars, and for general large language models, the training cost falls between US $2 million and US $12 million [7]. OpenAI obviously found the right business model to *share* such costs via, for example, a monthly user subscription, so there are strong reasons to believe that ChatGPT will survive well into the foreseeable future. In addition, Microsoft, the big investor behind OpenAI, recently announced a series of Office products that will be deeply integrated with ChatGPT for AI-assisted productivity improvement. OpenAI also launched its own application store, and it allows connection to >5000 other applications via the Zapier ChatGPT plug-in. ChatGPT looks unstoppable.

There have been various concerns about AI since its concept was consolidated back in the 1950s. Many people deem ChatGPT as the first genuine and universal AI product; it is thus not surprising that a wide variety of concerns have been raised about ChatGPT, especially given its popularity and versatility. One such important question is what jobs will be replaced by ChatGPT soon. Interestingly, ChatGPT itself can answer this question, although this does not help to relieve the concern. According to the many such answers available online, ChatGPT is likely to replace many jobs for which frequent human-to-human interactions are replaceable or not necessarily required, such as customer service representatives, translators, entry-level clerks, telemarketers, tutors, and virtual assistants. ChatGPT is also good at certain advanced tasks that are traditionally performed by domain experts. For instance, as this editorial is being written, several PhD students under our supervision are using ChatGPT for computer coding, data analysis, and even theoretical proof. It turns out that ChatGPT can build a pipeline for vital sign signal (eg, electrocardiogram, electroencephalogram) preprocessing in 3 seconds, which usually takes months to train a PhD student to accomplish. People are also experimenting with ChatGPT-assisted content development such as paint, video games, and movies. The impact of ChatGPT on our job market is real.

How is ChatGPT changing nursing and health care education? Trained on big data, ChatGPT is probably more knowledgeable than many human instructors in almost every discipline, especially on basic- to moderate-level topics. Additionally, remember that ChatGPT is an evolved form of a search engine: it can locate knowledge new to itself when necessary. ChatGPT is as competent and self-motivated as an omniscient human instructor in many senses; therefore, it is not surprising if ChatGPT becomes a component of the nursing education system in the near future, assuming health care policy makers and educators are not against such AI technologies. Even if this scenario does not happen soon, it is hard to believe that people will not use ChatGPT as a tutor or for self-teaching. In short, ChatGPT will play its role in education sooner or later. However, multiple institutions/divisions/departments/individuals have banned the use of ChatGPT by students in, for example, article writing and homework assignments. While the underneath ethic concerns (eg, on plagiarism) are completely legitimate, the ban of ChatGPT may not last long for many reasons, one of which is that Microsoft is integrating ChatGPT into their prevailing Office products under the name brand Microsoft 365 Copilot. Our educators and policy makers may need to rethink and reshape our education system by allowing students to use ChatGPT as a learning assistant.

The impact of ChatGPT on the nursing and interdisciplinary research ecosystem is profound. For instance, a recent study presented AI-enhanced protein design and discovered proteins that never existed before [8]. We also witnessed the recent use of ChatGPT in various scientific questions like intelligent transportation [9]; drug discovery [10]; and nursing education, research, and practice [11]. Some interesting questions include whether researchers who know better about AI technologies and have more access to AI tools will be able to do better research in terms of, for example, productivity and quality. Additionally, if AI technology becomes the biggest determining factor in research, how should we evaluate human researchers’ contributions? Will it become more difficult for junior researchers to establish their own independent research programs? How should we train the next generation of multidisciplinary researchers and scientists?

Finally, ChatGPT’s competitors like Google Bard should be mentioned to avoid the impression that ChatGPT is the single AI tool dominating our world. It is of significant interest to observe whether OpenAI’s and Google’s AI products have different *personalities* and capacities, thus rendering more human diversity and creativity.

## Abbreviations

AI: artificial intelligence
